# Repaired Tetralogy of Fallot: Have We Understood the Right Timing of PVR?

**DOI:** 10.3390/jcm13092682

**Published:** 2024-05-02

**Authors:** Benedetta Leonardi, Marco Perrone, Giuseppe Calcaterra, Jolanda Sabatino, Isabella Leo, Martina Aversani, Pier Paolo Bassareo, Alice Pozza, Lilia Oreto, Sara Moscatelli, Nunzia Borrelli, Francesco Bianco, Giovanni Di Salvo

**Affiliations:** 1 Bambino Gesù Children’s Hospital, IRCCS, 00165 Rome, Italy; 2 Clinical Pathways and Epidemiology Unit, Bambino Gesù Children’s Hospital, IRCCS, 00165 Rome, Italy; marco.perrone@opbg.net; 3 Division of Cardiology and CardioLab, Department of Clinical Sciences and Translational Medicine, University of Rome Tor Vergata, 00133 Rome, Italy; 4 Department of Cardiology, University of Palermo, 90133 Palermo, Italy; peppinocal7@gmail.com; 5 Department of Experimental and Clinical Medicine, Magna Graecia University, 88100 Catanzaro, Italy; jolesbt@hotmail.it (J.S.); isabella.leo98@gmail.com (I.L.); 6 Paediatric Cardiology and Congenital Heart Disease, University of Padua and Pediatric Research Institute (IRP), Città Della Speranza, 35127 Padua, Italy; martiaavesani1@gmail.com (M.A.); giovanni.disalvo@unipd.it (G.D.S.); 7 School of Medicine, University College of Dublin, Mater Misericordiae University Hospital, D07 R2WY Dublin, Ireland; piercard.dublin@gmail.com; 8 Dipartimento di Medicina Clinica e Sperimentale, Università di Messina, 98122 Messina, Italy; liliaoreto@hotmail.com; 9 Institute of Cardiovascular Sciences University College London, London WC1E 6BT, UK and Centre for Inherited Cardiovascular Diseases, Great Ormond Street Hospital, London WC1N 3JH, UK; sara.moscatelli90@gmail.com; 10 Adult Congenital Heart Disease Unit, AO Dei Colli, Monaldi Hospital, 80131 Naples, Italy; nunziaborrelli16@gmail.com; 11 Cardiovascular Sciences Department, AOU “Ospedali Riuniti”, 60126 Ancona, Italy; francesco.bianco@ospedaliriuniti.marche.it

**Keywords:** tetralogy of Fallot, PVR, pulmonary insufficiency

## Abstract

Despite many advances in surgical repair during the past few decades, the majority of tetralogy of Fallot patients continue to experience residual hemodynamic and electrophysiological abnormalities. The actual issue, which has yet to be solved, is understanding how this disease evolves in each individual patient and, as a result, who is truly at risk of sudden death, as well as the proper timing of pulmonary valve replacement (PVR). Our responsibility should be to select the most appropriate time for each patient, going above and beyond imaging criteria used up to now to make such a clinically crucial decision. Despite several studies on timing, indications, procedures, and outcomes of PVR, there is still much uncertainty about whether PVR reduces arrhythmia burden or improves survival in these patients and how to appropriately manage this population. This review summarizes the most recent research on the evolution of repaired tetralogy of Fallot (from adolescence onwards) and risk factor variables that may favor or delay PVR.

## 1. Introduction

The surgical repair of tetralogy of Fallot (rToF) in neonatal age often involves pulmonary valvotomy, a transannular patch, or incision in the right ventricular (RV) infundibulum, all of which can contribute to long-term pulmonary valve (PV) regurgitation. Chronic pulmonary regurgitation (PR) leads to right ventricular (RV) dilatation and/or dysfunction, left ventricular (LV) dysfunction, and possible life-threatening ventricular arrhythmias [1,2,3]. RV dilatation and/or dysfunction may also be present as early as the age of 10–18 years old, while ventricular arrhythmias appear to occur late in the second/third decade of life [4,5]. Although the majority of rToF patients with severe RV dilation and normal function/initial minimal reduction in function report being asymptomatic, the scientific community has advocated for early PV replacement, in order to eliminate chronic ventricular overload and prevent long-term adverse cardiac events [6,7].

Progressive RV dilatation and/or dysfunction and reduced LV ejection fraction (EF) have been identified as key risk factors for ventricular arrhythmias [8,9]. End-diastolic and end-systolic RV volume cut-offs have been associated with a higher RV size reduction after PVR, based on the hypothesis that it improves outcomes.

Thus, RV end-diastolic and end-systolic volumes have been set at 150–170 and 80–85 mL/m^2^ in recent years, respectively, and PVR is advised once these values are reached [10]. These values were determined in hopes that restoring PV competence could prolong patient lifespan as well as improve RV function [7,11,12,13].

However, the precise time in which the patient’s unfavorable ventricular remodeling will develop is unknown.

Even in clinical practice, a progressive significant worsening of RV dilatation and/or RV dysfunction during follow-up in the majority of the patients is not commonly seen [14,15], even in those with a considered severe RV dilatation (150 mL/m^2^). Therefore, even in patients with severe RV dilatation (150 mL/m^2^) with preserved ventricular function, the “external” change in the right ventricle can be very slow and almost imperceptible. The patient could remain in an apparent state of well-being for a further 2–3 years or perhaps more, and the reduction in RV EF over time can be preserved. In addition, the decrease in RV EF could not be an ideal and effective marker for disease progression [16]. In fact, the RV EF is load-dependent and reflects the ventriculo-arterial coupling rather than contractility [16]. On the contrary, we are not aware whether this state of continuous volumetric overload, leading to certain dilations of the RV, is associated with the progression of “imperceptible” myocardial fibrosis (undetectable by LGE), which can only be evaluated by the RV extracellular volume fraction using cardiac resonance imaging [3]. The RV extracellular volume fraction correlates with the histologic collagen volume fraction and can also be altered in patients with a “normal” delayed enhancement compatible with surgery performed in childhood. Moreover, it has been shown to be linked to cardiac adverse events in symptomatic adult rToF patients [17] and, therefore, should be used more in clinical practice. In fact, if we are focused only on progressive RV dilation, the majority of studies existing in the literature reported only a small increase in average RV volumes over time, which could be compatible with the expected CMR intra- and interobserver variability for RV volumes in an individual patient [18]. Only 10–15% of rToF patients experienced significant progressive RV changes that absolutely need to be treated [15].

**Figure 1:**
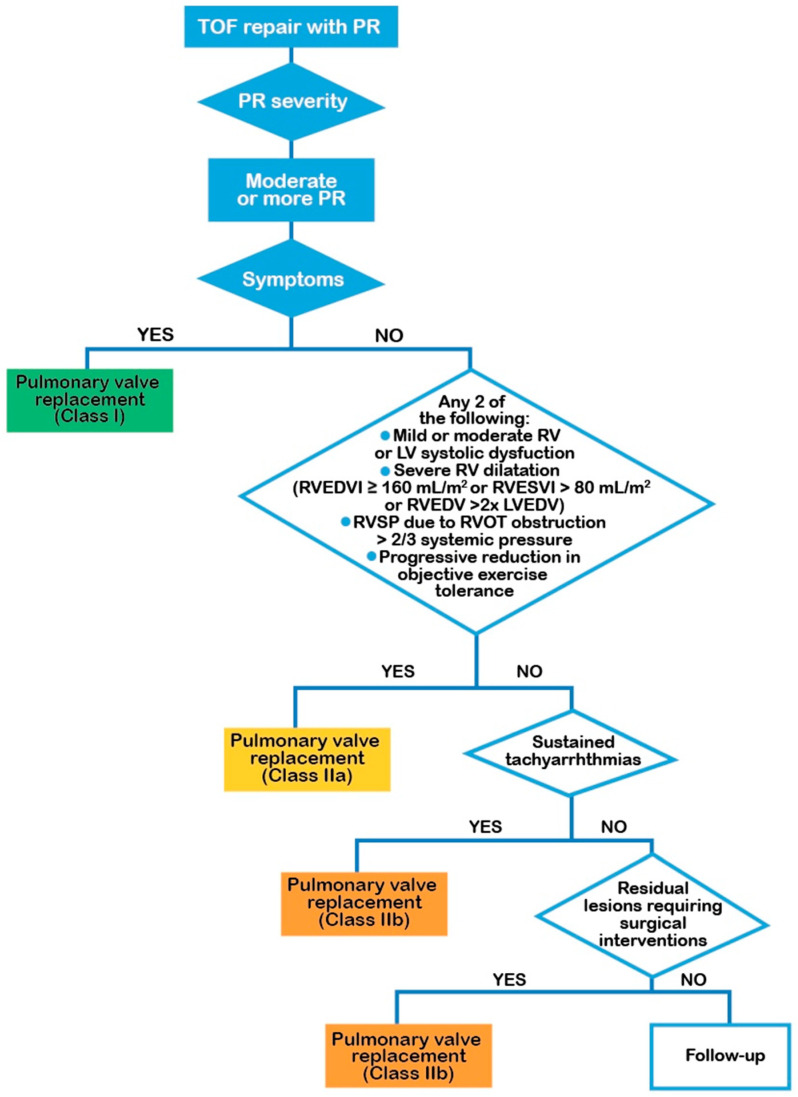
Adapted from [7]. Legend: LV = left ventricle; LVEDVI = left ventricular end diastolic volume indexed for BSA; PR = pulmonary regurgitation; RV = right ventricle; RVEDVI = right ventricular end-diastolic volume indexed for BSA; RVESVI = right ventricle end-systolic volume indexed for BSA; RVSP = right ventricle systolic pressure; right ventricle outflow obstruction. TOF, tetralogy of Fallot. Symptoms may include dyspnea, chest pain, and/or exercise intolerance referable to PR or otherwise unexplained.

In the remaining asymptomatic patients, there is still no consensus on the RV end-diastolic volume index (EDVi) cut-off at which RV dysfunction occurs, when PVR can entirely revert RV dilation and the adverse RV remodeling due to volumetric overload. In fact, it is not clear whether “microscopic” fibrosis in rToF patients arises early on at birth or even in the fetus and worsens incessantly throughout the patient’s life although the patient has been undergone PVR, once the disease process has started. We still have to understand whether PVR can determine complete reverse remodeling or if it may just slow down the progression. In addition, we still need to understand whether there is a threshold for fibrosis severity beyond which there can be no more complete reverse remodeling [19]. The latter questions are of fundamental importance, given that we do not know what “range” of right ventricular dimension recovery after PVR coincides with a significant reduction in fatal ventricular arrhythmias. Cools et al. have demonstrated that PVR improves the presence of the fibrotic response to PR, regardless of timing, once again posing the unanswered question about the correct timing for PVR [20]. Majeed et al. documented that when controlling for pre-CMR PVR status, the RV EDVi was not independently associated with the outcomes [21]. Pastor et al. suggests that RV volumes after PVR were associated with adverse clinical outcomes in rToF patients [22]. However, the question is not about whether PVR is associated with reversible RV remodeling, RV improvement, and notable symptomatic benefits in the mid-term follow-up, which has already been demonstrated in various papers [23], but rather when to conduct it. Van den Eynde et al. [24] in a recent meta-analysis suggested that the indications for asymptomatic rToF patients are restricted to the following situations: a decrease in objective exercise capacity, progressive RV dilation, progressive RV systolic dysfunction, progressive tricuspid regurgitation (at least moderate), RV outflow tract obstruction with RV systolic pressure exceeding 80 mmHg (tricuspid regurgitation velocity > 4.3 m/s), and sustained atrial/ventricular arrhythmias (class IIa) concluding that optimal timing of PVR remains challenging. Recently, Bokma et al. have documented the benefits of PVR in the long-term on a very broad international rToF cohort, defined as freedom from death and sustained ventricular tachycardia [25]. One more time, we have to consider that the benefits from PVR were the most pronounced in patients with advanced disease. The latter were considered rToF patients with right end-diastolic and end-systolic volumes of >180 and >95 mL/m^2^, respectively, RV EF < 40%, LV EF < 45%, and QRS duration >180 ms; then, the most significant benefits from PVR were documented in the patients with worse RV dilation and dysfunction than that considered by the guidelines to indicate PVR (Figure 1). In addition, even though Bokma et al. showed some benefits of PVR in rToF patients having “proactive” criteria (RVEDVi > 160 mL/m^2^, RVESVi > 80 mL/m^2^, RV EF < 47%, LV EF < 55%, QRS duration > 160 ms), it is not clear whether these patients were symptomatic or not and what the real benefit was. In fact, the same authors concluded that the purpose of the paper was not to determine the precise threshold for PVR in rToF and that a “prophylactic” approach of implanting a pulmonary valve in all asymptomatic patients with PR may be associated with a higher rate of adverse events [25].

Despite those conflicting results, the above-mentioned RV parameters assessed by cardiac magnetic resonance associated with a significantly wider QRS duration usually push clinicians to opt for PVR, even in asymptomatic patients. If we take into consideration that a significant number of rToF patients do not exhibit symptoms during adolescence, or at least until the end of their second decade of life, it is noteworthy that a considerable number of these patients have already undergone PVR.

In addition, even using electrocardiographic monitoring, significant arrhythmias are rarely detected in the first two decades of life [4,26]. Furthermore, one of the major causes of arrhythmias, the surgical scar created during neonatal repair, remains, even if the RV volume overload is removed. Therefore, it has been suggested that PVR could reduce but does not eliminate potentially fatal ventricular arrhythmias in this population [19,27], not to mention the fact that biological prostheses have a very limited lifespan. Indeed, the existing biological valves were not initially designed for young teenagers. Rather, they were primarily developed for elderly individuals, who present a distinct set of challenges. It is expected that all patients who have undergone prosthetic PVR will experience eventual graft failure, necessitating subsequent surgical or interventional procedures. The potential for heightened risk associated with each subsequent repeat treatment, such as infective endocarditis, must be considered during the initial decision-making process regarding PVR, especially in cases involving asymptomatic individuals.

The unresolved issue in these patients pertains to a significant proportion of individuals who exhibit a right ventricular dilatation of approximately 150–160 mL/m^2^ and preserved function. Notably, these patients not only subjectively report being asymptomatic but they also demonstrate comparable functional capacity to those with minor dilations of the right ventricle. O’Meagher et al. [28,29] had already highlighted that young adults (age at evaluation 26.2 + 8.8 years) with rToF and severe RV dilatation could have a similar oxygen consumption rate as those with less dilatation. This study, although performed on a small population (55 patients), the majority with severe RV end-diastolic volume (>150 mL/m^2^) and a high level of physical activity, had already questioned the common clinical practice of overvaluing RVEDV for the indication of PVR. Therefore, we believe that we should give less importance to the RV dimension (obviously within certain limits—about 170–180 mL/m^2^) when the patient is young and with a preserved biventricular function given that in many patients, the dilated RV allows to provide sufficient augmentation of cardiac output to meet exertional demands. In order to bridge the uncertainty regarding the patient identification of asymptomatic rToF patients with severe dilation who are at risk of sudden cardiac death or right ventricular failure, the best option would perhaps be an accurate detection of the interval reduction in RV systolic function in these patients subdividing them on the basis of ranges of severe right ventricular dilatation. Consequently, there is a need for a distinct therapeutic approach in these cases.

## 2. Parameters to Be Considered in Repaired ToF (Figure 2)

### 2.1. RV Size, RV Systolic Dysfunction, LV Dysfunction

It is well known that chronic PR leads to RV dilatation, which consequently leads to the dysfunction of the RV and then to the modification of LV geometry and function [30]. The duration of this condition as a whole, whether each change occurs within a specific time interval or whether LV involvement commences simultaneously with the progression of RV dysfunction, is still unknown. While Geva et al. [8] established that RV systolic dysfunction is independently linked to impaired clinical status in long-term rToF survivors, rather than RV diastolic dimensions, the question of whether RV dysfunction originates from dilatation continues to be unanswered. Additionally, there is still considerable uncertainty regarding the extent to which ventricular–ventricular interaction in patients with rToF influences the deterioration of LV function and whether there is a specific timing at which LV dysfunction initiates. In this regard, a possible answer could come from an accurate evaluation and quantification of septal motion, often paradoxical, that may demonstrate intraventricular dyssynchrony [31,32]. Additional concerns for rToF are the existence of an akinetic or aneurysmal segment of the RV outflow tract and the presence of a right ventriculotomy. These factors have been shown to have an adverse impact on RV EF in the late stages following ToF repair [9,33]. The issue of the onset of mono- or biventricular dysfunction in rToF in relation to significant RV dilation is of paramount importance given that a large percentage of patients have severe dilation without an “apparent” impairment of function for many years during adolescence/the second decade of life. Furthermore, in this population, it is fundamental to prevent RV dysfunction and consequently LV impairment, as ventricular dysfunction has been recognized as one of the main causes of sudden death [21,34,35]. Bokma et al. in an international ToF multicenter registry (INDICATOR) including 575 rToF adults (31 ± 11 years old) documented that, in addition to RV dysfunction, RV hypertrophy is also an important risk factor associated with poor clinical outcomes in rToF patients [34]. In fact, rToF patients with CMR-derived RV EF ≥ 42% and an RV mass index < 39 g/m^2^ were at low risk for major adverse clinical outcomes after 50 years of age. Recently, the prognostic role of RV hypertrophy has been repeatedly reconfirmed [21,36,37,38]. In the contemporary literature, there has been a growing body of research exploring the correlation between speckle-tracking analysis measured through echocardiography and both RV and LV EF as assessed through CMR [39,40,41,42,43]. Speckle-tracking analysis offers several clinical implications for the management of rToF patients [39,43,44,45]. Traditionally, the assessment of ventricular function in rToF patients has relied on measures such as an ejection fraction, which may not fully capture subtle changes in myocardial mechanics. Speckle-tracking analysis, on the other hand, provides a more comprehensive evaluation of myocardial deformation, including strain, strain rate, and torsion. By quantifying these parameters, speckle-tracking analysis can detect early signs of myocardial dysfunction before overt ventricular impairment becomes clinically apparent. This early detection is particularly crucial in rToF patients, who may experience progressive deterioration in ventricular function over time. As proof of this assumption, speckle-tracking parameters have been shown to correlate with clinical outcomes in rToF patients [44,46]. Several studies have reported associations between abnormal strain patterns and adverse events such as heart failure, arrhythmias, and sudden cardiac death [36,46,47]. Specifically, decreased longitudinal strain and increased myocardial dys-synchrony have been identified as independent predictors of adverse outcomes in rToF patients offering the possibility of highlighting those at higher risk for complications. Additionally, speckle-tracking analysis can aid in the optimization of therapeutic strategies by providing objective measures of myocardial function. For instance, it can help guide decisions regarding the timing of surgical interventions, such as PVR [32,48]. In clinical practice, certain cut-offs derived from speckle-tracking analysis can be considered during follow-up evaluations of rToF patients, indicating subclinical ventricular dysfunction and therefore an increased probability of adverse events below certain values [37,46,49]. Torsion, another parameter assessed by speckle-tracking analysis, can also provide valuable insights into ventricular mechanics and may be useful for risk stratification in rToF patients [50,51,52].

Finally, in addition to the above-mentioned techniques, the systolic excursion of the tricuspid annulus (TAPSE), the fractional shortening of the area of the RV (FAC), and three-dimensional echocardiography (RT3DE), knowing its limits, still remain useful in the routine evaluation of rToF patients [40,53,54] as the screening tool to highlight an initial problem of ventricular dysfunction.

### 2.2. Diastolic Dysfunction

In rToF, diastolic dysfunction as measured by cardiac catheterization and echocardiographic indices has demonstrated clinical outcome associations including arrhythmia and decreased exercise capacity [55,56,57]. Kikano et al. documented that adolescent and adult rToF subjects have abnormal LV diastolic function compared to healthy controls [56]. The indices of LV diastolic function were associated with arrhythmia and mortality [56]. Aboulhosm et al. showed that Doppler indices indicative of RV and LV diastolic dysfunction correlated with an increased presence of ventricular arrhythmia in an adult rToF population (36.8 ± 12 years) [55]. Van den Berg documented abnormalities in RV filling in 36 young rToF patients (median age at study inclusion, 17 years), which included impaired relaxation and signs of restriction of RV filling [58]. In addition, invasive measurements in rToF have demonstrated increased RV end-diastolic pressure in a consistent percentage of patients [59,60]. Egbe et al. also showed that severe RV diastolic dysfunction assessed by cardiac catheterization is an independent risk factor for death/transplant in the third decade of life of these patients [59]. In the same study, the authors also found that inferior vena cava dilatation and hepatic vein diastolic flow reversal had the best sensitivity and specificity for detecting severe RV diastolic dysfunction, defined as an RV end-diastolic pressure > 14 mmHg or RA pressure > 10 mmHg [59]. However, the role of RV restrictive filling, per se, is still debated given that demonstrating the presence of diastolic dysfunction is complicated by the lack of reliable markers. In fact, the right ventricular filling parameters are influenced by age, heart rate, and the presence of left ventricular diastolic dysfunction and show wide variability with the act of breathing. The investigation of right atrial function with deformation imaging, both with echocardiography and CMR, may be promising in the assessment of RV diastolic function, although this is still poorly characterized.

### 2.3. Right Ventricular Fibrosis

Late gadolinium enhancement (LGE) imaging is used in CMR investigations to reveal repair-induced cardiac fibrosis [61,62,63]. Repaired ToF patients can have fibrosis in the RV at surgical sites in the outflow tract, ventricular septal defect patching, and inferior insertion point. Fibrosis can also be seen in the RV’s trabeculated myocardium, apex, inferior or lateral wall, and left ventricle infarction, and there is usually not significant progression in the extent of RV LGE at the intermediate term follow-up of these patients [62]. Therefore, the assessment of LGE is of fundamental importance in rToF patients, given that it has long been linked to malignant tachyarrhythmias and sudden cardiac mortality in rToF as well as to exercise intolerance [61,62,63,64,65]. However, given recent concerns regarding the safety of gadolinium-based contrast agents, frequent assessment of LGE may not be necessary in follow-up once it has been properly assessed, unless there is a significant worsening of ventricular function and/or the patient’s clinical status. In fact, Saengsin et al. has shown that the RV LGE extent and LGE score did not increase over time after rToF repair in 127 patients (median age at first CMR 18.9 years) who had at least two CMRs (a median follow-up duration of 4.0) [66]. Two-dimensional (2D) LGE is most used in clinical practice, although the spatial resolution of such methods may be insufficient to accurately characterize the thin-walled RV wall, and the robustness of semi-quantitative approaches may be sub-optimal. In fact, 2D LGE does not have the entire contiguous coverage of the heart enabling the precise volumetric quantification of the total LGE burden and have a more minor spatial resolution noninvasive scar definition than three-dimensional (3D) LGE CMR [67]. Ghonim et al. recently performed a study in sixty-nine adults with rToF (mean age 40 ± 15 years) who underwent VT stimulation. It showed that 3D LGE CMR-defined scar burden can predict inducible VT with a high sensitivity and specificity for an RV LGE volume of 25 cm^3^ [64]. They also highlighted that a small increase in LGE volume is associated with a large increase in the risk of inducible VT whereby for every 1 cm^3^ of LGE, there is a 15% increased risk of inducible VT [64]. Ghonim et al. also suggested that the 3D LGE extent is strongly associated with inducible VT and is stronger than established predictors, including age, QRS duration > 180 ms, and non-sustained VT. Although the evidence in the literature has unquestionably demonstrated the correlation between the presence of LGE and adverse cardiac events in this population, recently, the importance of diffuse myocardial fibrosis in this pathology is also emerging to stratify the risk of adverse events. In fact, the recent improvements in CMR, such as myocardial T1, have revealed diffuse myocardial fibrosis that LGE missed [68,69]. Myocardial T1 values quantify the extracellular volume fraction (ECV), which correlates with the histologic collagen volume fraction. Specifically, ECV reflects the ratio of the extracellular matrix volume to the total myocardial volume (extracellular matrix volume plus cardiomyocyte volume), and increased ECV indicates either that the expansion of the extracellular matrix exceeds cardiomyocyte hypertrophy or cardiomyocyte atrophy/death while the extracellular matrix component becomes proportionally larger. Therefore, we can hypothesize that in rToF patients with volume overload, the increasing RV ECV, if combined with a decrease in the RV mass-to-volume ratio, as documented in Chen et al.’s study [68], could be consistent with a maladaptive process at the cellular level characterized by cardiomyocyte atrophy and diffuse fibrosis. In fact, ECV CMR markers of diffuse fibrosis do not seem to be influenced by focal scar burden and show different functional correlates, rather related to RV dilatation and to the degree of PV regurgitation than to systolic dysfunction [68,70,71]. The presence of diffuse fibrosis in rToF has been shown to correlate to adverse events in this population [17,38,68,72]. In 84 rToF patients (median age 23.3 years), Chen et al. found that increased ECV was associated with RV volume overload and arrhythmia [68]. LV and RV ECV values were positively correlated, suggesting that interstitial remodeling in rToF could be induced by diffuse signaling pathways involving both the right and left ventricles. However, while an LV ECV above the upper limit of normal (>28%) was independently associated with arrhythmia, RV ECV was similar in arrhythmia and non-arrhythmia patients [68]. On the contrary, Shiina et al., in a cohort of symptomatic adults (35.4 ± 13.8 years old) in New York Heart Association class 2–4, documented RV ECV in addition to septum ECV as predictors of adverse events [17]. Adults with septum ECV > 29.0% and RV EF < 45% were more likely to experience cardiac events. However, the definition of arrhythmia, age of patients, and study designs are different in Chen and Shiina’s papers [17,68]. In addition, we should highlight the technical difficulty of T1 mapping and the poor reproducibility of T1 performed at the RV and LV level compared to that performed at the septal level. Cochet et al. found that patients with past PVR had shorter RV and LV native T1 values than those without PVR, suggesting that PVR may reduce the diffuse fibrosis [72]. These data could be important, because conventional parameters such as the RV EF are probably not enough to document potential myocardial impairments. Therefore, the combined ECV and RV EF could be a useful adjunct parameter to understand long-term PVR in these patients. Cools et al. have shown that PVR can attenuate (but not normalize) fibrosis in rToF patients, regardless of timing [20]. In fact, the macroscopic extent of RV reverse remodeling (as measured by ventricular volumes, mass, and the tricuspid regurgitation fraction) was similar after early and late PVR strategies, although the measures did not completely normalize compared to controls. Microscopically, PR enhanced interstitial fibrosis and collagen cross-linking, which decreased after early and late PVR treatments. Yuan et al. compared the degree of histological myocardial fibrosis at the biopsy of the right ventricular outflow tract tissue taken during PVR to follow-up after PVR in 51 rToF patients who underwent surgical PVR [73]. Histological myocardial fibrosis was linked to biventricular systolic functions. Higher myocardial fibrosis and older age at PVR independently predicted unfavorable cardiac events in rToF patients [73]. Severe RV fibrosis, evaluated by an RV sample obtained during PVR, was associated with an increased RV end-systolic volume index, RV mass, and RV area post-PVR in rToF [74]. In the severe fibrosis group, Yamamura et al. identified a trend toward increased heart failure events [74].

Although biventricular ECVs can be very informative, the cut-off value depends on the T1 mapping method and CMR conditions and ECV-LV and ECV-RV free wall are more motile and magnetically susceptible in comparison to septum ECV and consequently less reproducible. Therefore, the issue concerning fibrosis in ToF is still under debate. Interstitial fibrosis reactive to adverse loading should be distinguished from replacement fibrosis following scarring, patch material, and myocardial ischemia [16]. It is one of the factors that underlies myocardial dysfunction over time; however, it is not easily diagnosed in clinical practice. Therefore, given that fibrosis in rToF is multifactorial (focal and diffuse), with LGE we only see part of the problem, the one inherent to the consequence of reparative surgery and residual lesion. In fact, in the study by Chen et al., although LGE was present in 94% of the patients, most commonly in the RVOT (87%) and in the inferior septal–free wall junction (81%), the RV LGE score was not associated with RV ECV [68]. Focal and diffuse fibrosis are problems in ToF that seem to not be connected but that the degree of expression of each of them in the individual patient can make a difference in the long-term follow-up. Finally, we must also consider that fibrosis could be already present in both ventricles at the time of surgical repair, suggesting that the remodeling process begins early and continues over time. This also implies that the volume overload after surgical repair is only an aggravating factor. Unfortunately, we still do not know how the volume overload impacts the pre-existing disease, how the initial remodeling process underlying fibrosis evolves, or by which factors it is influenced over time. Finally, all surgeries that improve the overload, whether pressure or volumetric, can improve cardiac function but not definitively cure the myocardial pathology in ToF patients.

### 2.4. QRS Enlargement

QRS prolongation or enlargement may develop years after successful ToF surgery. Some investigations have highlighted RV enlargement secondary to pulmonary regurgitation as the major determinant of QRS prolongation at surface ECG [75,76,77]. Prolonged QRS duration has been linked to larger right and left ventricles and poorer function as well as to heterogeneous ventricular mechanical activation and reduced lateral and septal left ventricle wall strain [78]. In addition, some studies have identified QRS duration >180 ms and older age at repair as predictors of sudden cardiac death (SCD) [79,80,81]. Valente et al. [36] found that QRS duration ≥ 180 ms alone was a weak predictor of death or sustained ventricular tachycardia, but adding the CMR-measured RV mass-to-volume ratio and EF significantly improved the outcome prediction in a multicenter study involving 871 ToF patients. The increase in QRS duration by 0.6 msec/year seems to lead to VT development, mostly after 20 years following surgery [82]. Repaired ToF patients with greater QT and JT dispersions and a QRS ≥ 180 ms are at higher risk for VT, indicating that both depolarization and repolarization anomalies are linked to VT. In the long-term follow-up of ToF patients after PVR, substantial QRS prolongation and no QRS duration reduction have been indicated as important risk factors [83,84]. Scherptong et al. [84] reported that a post-PVR QRS duration exceeding 180 ms was associated with a reduced freedom from a composite end point including death, re-PVR, ventricular tachyarrhythmia, and symptomatic heart failure after 5 years. Chaaya et al. showed that increased age at PVR, a QRS ≥ 180 ms post-PVR, no reduction in QRS after PVR, and a history of VT were associated with higher risk of VT, ICD shock for sustained VT, or inducible VT on EP studies in a study involving 85 adult rToF patients (median follow-up 3.6 years; median age 34 years) [83]. The change in QRS was linearly correlated with the change in the RVEDVi, and a QRS ≥ 150 ms after PVR had a 100% sensitivity and 40% specificity for detecting VT outcome in their study [83]. Therefore, although there is still no consensus regarding whether PVR can decrease QRS duration and to what extent this corresponds to a significant reduction in risk [83,85,86,87,88], it is reasonable to state that older rToF patients with prolonged QRS which is not modified after PVR warrant closer monitoring. On the other hand, the predictivity of QRS duration beyond certain values of the spontaneous unfavorable arrhythmic events and electrophysiological inducible VT in rToF patients has already been highlighted in the literature by Horwich et al. in 2003 [89]. They also mentioned the fact that it acquires even more importance in the presence of other risk factors well known in this population (older age, significant LGE, transannular repair with transventricular approach, ventricular dysfunction).

**Figure 2:**
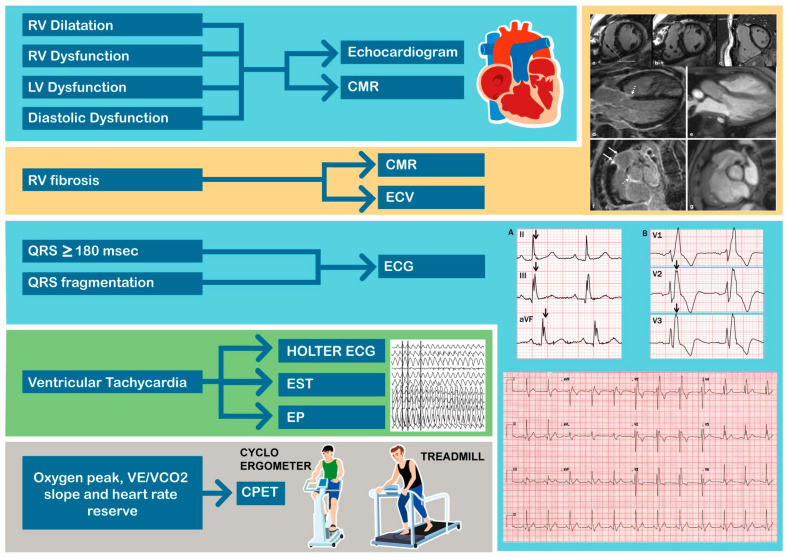
The parameters to be considered in repaired ToF. CMR = cardiac magnetic resonance; CPET = cardiopulmonary exercise test; ECV = extracellular volume fraction; EST = exercise stress test; EP = electrophysiological study; LV = left ventricle; RV = right ventricle. ECG: (**A**). Inferior fragmentation (black arrows) in a patient with QRS < 120 msec. (**B**). Anterior fragmentation (black arrows) in another patient with presence of right bundle branch block.

### 2.5. QRS Fragmentation

QRS fragmentation has been defined as an additional R wave (R’) or notch in the nadir of the S wave in ≥2 contiguous leads (right-sided/septal: aVR, V1, V2; anterior: V2–V5; lateral: I, aVL, V5, V6; or inferior: II, aVF, III) in patients with QRS duration < 120 ms. In patients with right bundle branch block, QRS-f has been defined as ≥3 R waves/notches in the R/S complex (more than the typical 2 in right bundle branch block) in ≥2 contiguous leads. In patients with paced QRS and premature ventricular complexes, QRS-f was defined as ≥3 notches in the R/S complex [90]. QRS fragmentation has been linked to greater RV LGE scores, lower RV ejection fractions, bigger RV volumes, and RV outflow tract aneurysms in rToF patients [91,92]. Shanmugam et al. documented that an increased fragmentation burden, especially in the anterior leads, is associated with increasing RV dysfunction [91]. In a case series of 37 adults with rToF, Park et al. found that QRS fragmentation was associated with more extensive RV fibrosis and dysfunction as detected on CMR [92]. In the study by Bokma et al., in 794 adult patients with ToF, the severity of QRS fragmentation independently predicted all-cause mortality and ventricular arrhythmias [90]. QRS fragmentation predicted all-cause mortality better than QRS duration alone and indicated cardiac fibrosis [90]. This evidence has also been confirmed in another wide cohort of 465 rToF patients by Egbe et al., in which the severity of QRS-f was an independent predictor of all-cause mortality after adjustment for other ECG parameters, patient demographics, and atrial and ventricular arrhythmia. Interestingly, only QRS fragmentation predicted the optimal ICD therapy in the current long-term French Registry of ToF patients with ICDs [93]. QRS fragmentation was the only independent predictor of appropriate ICD therapies (hazard ratio, 3.47 [95% CI, 1.19–10.11]), and its integration in a model with current criteria increased the 5-year time-dependent area under the curve from 0.68 to 0.81 (*p* = 0.006). Therefore, this parameter should be considered in the periodic assessment of rToF patients.

### 2.6. Atrial and Ventricular Arrhythmias

Arrhythmia and sudden cardiac death remain common in repaired tetralogy of Fallot and affect even those with excellent anatomic repairs [26]. The most common arrhythmogenic mechanisms in rToF are surgical scars and natural conduction barriers that produce macro-re-entry [94]. Adult patients with rToF with advancing age are at risk of developing re-entrant atrial arrhythmias, including intra-atrial re-entrant tachycardia and cavo-tricuspid isthmus-dependent atrial flutter. Khairy et al. [95] in a multicenter study of 556 adult rToF patients (36.8+/-12.0 years) showed that sustained arrhythmias or an arrhythmia intervention is not an infrequent event in older patients. In fact, in the multicenter study, 43.3% of the population had a sustained arrhythmia or arrhythmia intervention [95]. The prevalence of atrial tachyarrhythmias was 20.1%. The factors associated with intra-atrial re-entrant tachycardia in multivariable analyses were right atrial enlargement (odds ratio [OR], 6.2; 95% confidence interval [CI], 2.8 to 13.6), hypertension (OR, 2.3; 95% CI, 1.1 to 4.6), and the number of cardiac surgeries (OR, 1.4; 95% CI, 1.2 to 1.6). Older age (OR, 1.09 per year; 95% CI, 1.05 to 1.12), lower left ventricular ejection fraction (OR, 0.93 per unit; 95% CI, 0.89 to 0.96), left atrial dilation (OR, 3.2; 95% CI, 1.5 to 6.8), and the number of cardiac surgeries (OR, 1.5; 95% CI, 1.2 to 1.9) were jointly associated with atrial fibrillation. Ventricular arrhythmias were prevalent in 14.6% and jointly associated with the number of cardiac surgeries (OR, 1.3; 95% CI, 1.1 to 1.6), QRS duration (OR, 1.02 per 1 ms; 95% CI, 1.01 to 1.03), and left ventricular diastolic dysfunction (OR, 3.3; 95% CI, 1.5 to 7.1). The prevalence of atrial fibrillation and ventricular arrhythmias markedly increased after 45 years of age. While right atrial size and the presence of at least moderate tricuspid regurgitation are associated with atrial arrhythmias, monomorphic ventricular tachycardia (the predominant form of VT in rToF patients) seems to be related to a surgically modified RV causing several macro-re-entrant routes [26]. Rhythmic disorders are more closely correlated to a focal scar on the septum and at other LV and RV locations than a scar on the RV outflow tract [72]. There are several risk factors for arrhythmia, including older patient age, older age at repair, previous ventriculotomy, older age at PVR, longer QRS duration (≥180 ms), QRS fragmentation, a greater RVEDVi, ventricular dysfunction, abnormalities in the global longitudinal strain and ventricular dys-synchrony, RV hypertrophy, and the presence and extent of LGE [34,37,47,57,79,90,95,96,97]. A meta-analysis of almost 7000 rToF patients found that older age at repair, prior palliative shunt, longer QRS length, and moderate RV dysfunction were linked with mortality and VT [97]. The risk factors for VT included older age (per 1 year, odds ratio [OR]: 1.039; 95% confidence interval [CI]: 1.025–1.053), older age at corrective surgery (per 1 year, OR: 1.034; CI: 1.017–1.051), previous palliative shunt (OR: 3.063; CI: 1.525–6.151), the number of thoracotomies (OR: 1.416; CI: 1.249–1.604), longer QRS duration (per 1 ms, OR: 1.031; CI: 1.008–1.055), and at least moderate right ventricular dysfunction (OR: 2.160; CI: 1.311–3.560). Additional risk factors for cardiac death/VT were a previous ventriculotomy (OR: 2.269; CI: 1.226–4.198), lower left ventricular ejection fraction (per 1%, OR: 1.049; CI: 1.029–1.071), and higher right ventricular end-diastolic volume (per 1 mL/m^2^, OR: 1.009; CI: 1.002–1.016). Supraventricular tachycardia/atrial fibrillation was an additional risk factor for all-cause mortality/VT (OR: 1.939; CI: 1.088–3.457) [97]. Among ToF patients referred for VT ablation, 15% have impaired LV systolic function and 35%-46% have moderate or severe RV function [98,99]. Beurskens et al. [100] found that RV systolic dysfunction, hypertrophy, dilatation, QRS prolongation, and obesity predict VT in rToF patients. Multivariable Cox hazards regression identified the right ventricular (RV) end-diastolic volume (hazard ratio [HR] 2.03, per 10 mL/m^2^ increase; *p* = 0.02), RV end-systolic volume (HR 3.04, per 10 mL/m^2^ increase; *p* = 0.04), RV mass (HR 1.88, per 10 g/m^2^ increase; *p* = 0.02), and RV ejection fraction (HR 6.06, per 10% decrease; *p* = 0.02) derived from CMR to be independent risk factors of VT [100]. VT development was most strongly linked to right ventricular dimensions (RVEDVI ≥ 160 mL/m^2^ and RVESVI ≥ 80 mL/m^2^) in a study by Rizt et al. including 434 rToF patients [101]. Bad cardiac events (symptomatic persistent VT, abortive SCD, SCD) happened 4.4% of the time. The median age at presentation with prolonged VT (63% of unfavorable cardiac events) was 24 years (18–37) [101]. In addition, a maximum right atrial area indexed to body surface area ≥16 cm^2^/m^2^ was found to independently predict clinically important ventricular arrhythmia, beyond atrial arrhythmia [102]. Finally, we should consider that although PVR usually results in a reduction in RV volumes and hemodynamic improvement, replacing the valve does not eliminate the anatomic substrate for monomorphic VT [85].

### 2.7. Oxygen Peak, VE/VCO2 Slope, and Heart Rate Reserve

A decreased peak oxygen consumption (VO2 peak ml/kg/min), the ratio of minute ventilation to carbon dioxide production (VE/CO2) slope, and heart rate reserve have been linked to morbidity and mortality in rToF patients [103,104,105,106,107]. A peak VO2 < 27 mL/kg/min has been associated with an increased risk of major adverse cardiac events [108]. While it is logical to assume that the VO2 peak would decline when PVR is needed, a diminished VO2 peak is not always present before PVR, and the precise VO2 peak values that are expected to serve as an indicator for PVR remain to be established. Although a cut-off of less than 70% predicted VO2 max has been identified to take PVR into consideration [109,110], the effect of pulmonary function on exercise-related symptoms must be considered in this patient population because cardiac surgery is unlikely to improve exercise capacity in a patient who is primarily pulmonary-limited to begin with [111]. In addition, it is not yet clear whether there is a real improvement in oxygen consumption after PVR [111,112,113,114]. Furthermore, it is imperative to establish a definitive differentiation between the VO2 peak cut-off for PVR observed prior to sexual maturity and the corresponding cut-off observed thereafter. Drawing a comparison between the oxygen peak of an adolescent and that of a young adult is seen inconceivable. Consequently, the optimal threshold for VO2 peak improvement following PVR is still not present in the latest guidelines for the management of patients with congenital heart disease [7]. Moreover, there is ongoing controversy regarding the effect of pulmonary regurgitation, RV dilatation, and/or RV EF on exercise capacity in these patients. While it is logical to assume that a higher degree of PR is associated with a diminished exercise capacity, it is widely recognized that both the volume and fraction of pulmonary regurgitation decrease significantly during exercise due to the elevated heart rate caused by exercise, which in turn shortens the diastolic time. Therefore, rToF patients who engage in regular physical activity may exhibit reduced RV volumes for a given volume of pulmonary regurgitation, in comparison to those who are less active but have the same quantity of PR. This mechanism could be one of the reasons why patients with significant RV dilations maintain a preserved functional capacity [28] and why in Rashid et al.’s [115] study no correlation emerged between the RV volume and PR fraction. Finally, the use of the VO2 max or predicted VO2 max as one of the parameters to give the indication for PVR in asymptomatic patients requires a profound knowledge of the patient, including their daily activity with at least two measurements over time.

## 3. Conclusions

The RV’s response over time shows a number of different patterns, and it is still a challenge to understand which one plays a major role in determining RV failure. The factors affecting ventriculo-arterial interactions, atrioventricular interaction, and interventricular interactions interfere with each other from patient to patient. Pulmonary valve replacement, which is not an ideal procedure, free from short-, medium-, and long-term risks, will be necessary for a sizable portion of rToF patients. The ideal PV substitute should be freely available, percutaneous, and simple to implant, fitting different RVOT anatomic substrates, not immunogenic, resilient to infections. When applied to younger people, the capacity for growth and long-lasting duration would be an extra attribute that would be desirable. At the moment, there is no such valve available. Thus, it remains a challenge to identify patients who are likely to benefit from PVR implanted at the right time, and this must be the current commitment of the entire world, given that this implies the survival of a significant proportion of patients with congenital heart disease. Until now, investigators have focused on measurements of biventricular volumes and function to guide the decision of PVR in asymptomatic patients, but these parameters ignore the composition and health of the myocardium. Many parameters and their limitations in relation to the feasibility and reproducibility, the patient’s age, and the patient’s lifestyle should be included in the evaluation of rToF patients. Those who manage such complex patients must have the knowledge of all existing techniques to understand what type of patient we are faced with and “adapt” the current knowledge and recommendations for PVR to the specific patient situation, considering that in such patients, there is no risk-free decision.

In addition, we must remember that PVR often does not revert ventricular dysfunction nor the widening of QRS complexes back to normal. Hence, the susceptibility to ventricular arrhythmias and sudden death remains an issue. Yet, this is the only procedure currently available to improve the RV overload and reduce LV dysfunction and should, therefore, be used expertly.
